# *Henryognathusthomasi*, a new genus and new species of *Arctostaphylos*-feeding plant bug from western North America (Miridae, Phylinae, Phylini)

**DOI:** 10.3897/zookeys.796.21432

**Published:** 2018-11-15

**Authors:** Randall T. Schuh, Ruth Salas

**Affiliations:** 1 Division of Invertebrate Zoology, American Museum of Natural History, New York, New York, USA 10024 American Museum of Natural History New York United States of America

**Keywords:** Arizona, California, Ericaceae, host plant, Oncotylina

## Abstract

*Henryognathus*, new genus, with the single included new species *H.thomasi*, is described from western North America. The taxon is recorded as feeding on species of *Arctostaphylos* (Ericaceae) in California and Arizona. Coloration and many morphological attributes are similar to species of *Plagiognathus* Fieber, but the structure of the male genitalia is distinctive.

## Introduction

Among the North American members of the subtribe Phylini: Oncotylina (Schuh and Menard 2013, Menard et al. 2014), the genus *Plagiognathus* Fieber is the most speciose, while having structurally rather homogeneous male genitalia and great similarity in many somatic features. Certain other taxa, however, are somatically similar to *Plagiognathus*, but possess distinctive male genitalia, including *Americodema* Henry, *Occidentodema* Henry, and *Lineatopsallus* Henry. In the present paper we describe an additional new taxon, *Henryognathusthomasi*, whose male genitalia are also distinct from the nearly 100 North American species placed in *Plagiognathus* by Schuh (2001), but which we also place in the Oncotylina.

## Dedication

This paper, and the new taxa described in it, are dedicated to Thomas J. Henry in recognition of his contributions to our knowledge of Miridae and Lygaeoidea. Through a combination of extensive fieldwork, coupled with faunistic, revisionary, and phylogenetic studies, Tom has advanced our understanding of true bug taxonomy and host associations. On behalf of all heteropterists who have had occasion to use the collections of the United States National Museum of Natural History, we recognize the contributions Tom has made to the organization, presentation, and content of the collections of that institution, bringing to light its status as one of the world’s truly great resources for the study of true bugs.

## Materials and methods

In total, 338 specimens were examined during the present study. “Unique specimen identifiers” (USIs), composed of an institution and project code (AMNH_PBI) and a unique number (00414919), were affixed to each specimen. Specimen data can be viewed on line through: research.amnh.org/pbi/heteropteraspeciespage/, discoverlife.org, and idigbio.org/portal. Measurements were prepared using a digital micrometer attached to a movable stage, the data being recorded directly to a spreadsheet; all measurements are in millimeters. Habitus images were prepared using a Microptics-USA/Visionary Digital photomicrographic system as developed by Roy Larimer; multiple layers were stacked using Helicon Focus software. Illustrations of the male genitalia were prepared as pencil drawings by using a Nikon Eclipse 80i microscope, then scanned and rendered using Adobe Illustrator.

The following institutional acronyms are used in the specimens examined section for specimen deposition:

**CNC** Canadian National Collection of Insects, Ottawa

**KU** Snow Entomological Museum, University of Kansas, Lawrence

**UCB** Essig Entomological Museum, University of California, Berkeley

**UCD** Bohart Entomological Museum, University of California, Davis

**UCR**University of California Entomological Research Museum, Riverside

**USNM** United States National Museum of Natural History, Washington, DC

**ZISP** Zoological Institute, Russian Academy of Sciences, St. Petersburg

## Taxonomy

### Henryognathus gen. n.

Taxon classificationAnimaliaHemipteraMiridae

http://zoobank.org/9A9F24C3-2E13-4225-9BBE-51862158DF78

#### Type species.

*Henryognathusthomasi*, new species.

#### Diagnosis.

**Figure 1.**
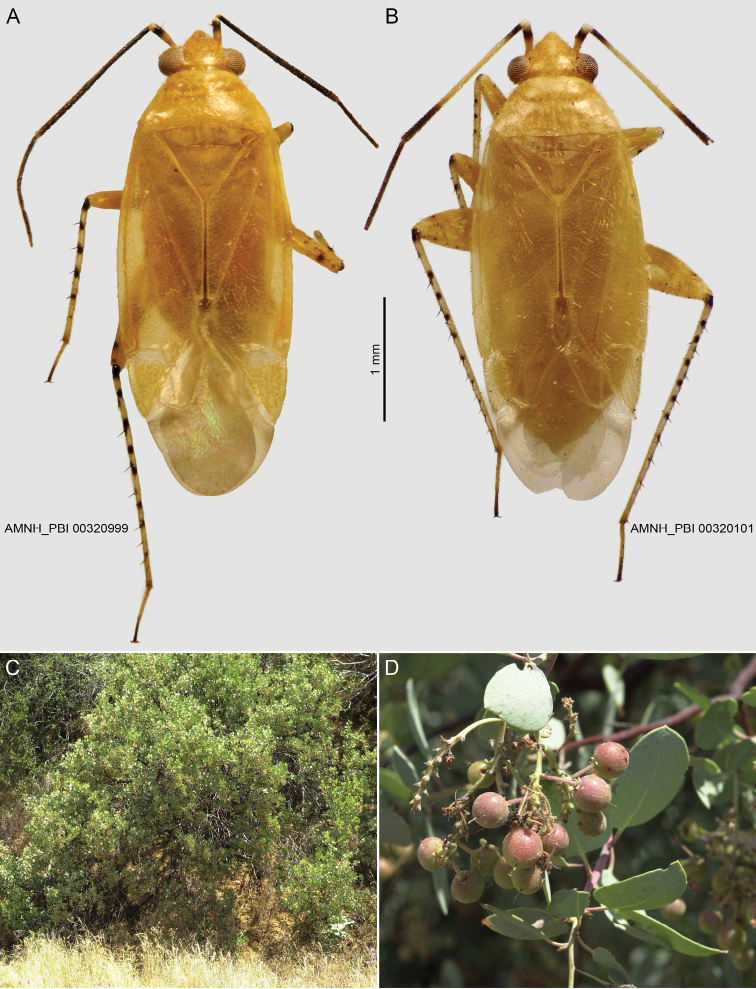
Habitus and hosts of *Henryognathusthomasi*. **A** Male **B** Female **C–D***Arctostaphylosviscida*, California: Tulare Co.: NE of Springville on Bear Creek Rd near Scicon, 36.21394°N, 118.7716°W.

**Figure 2.**
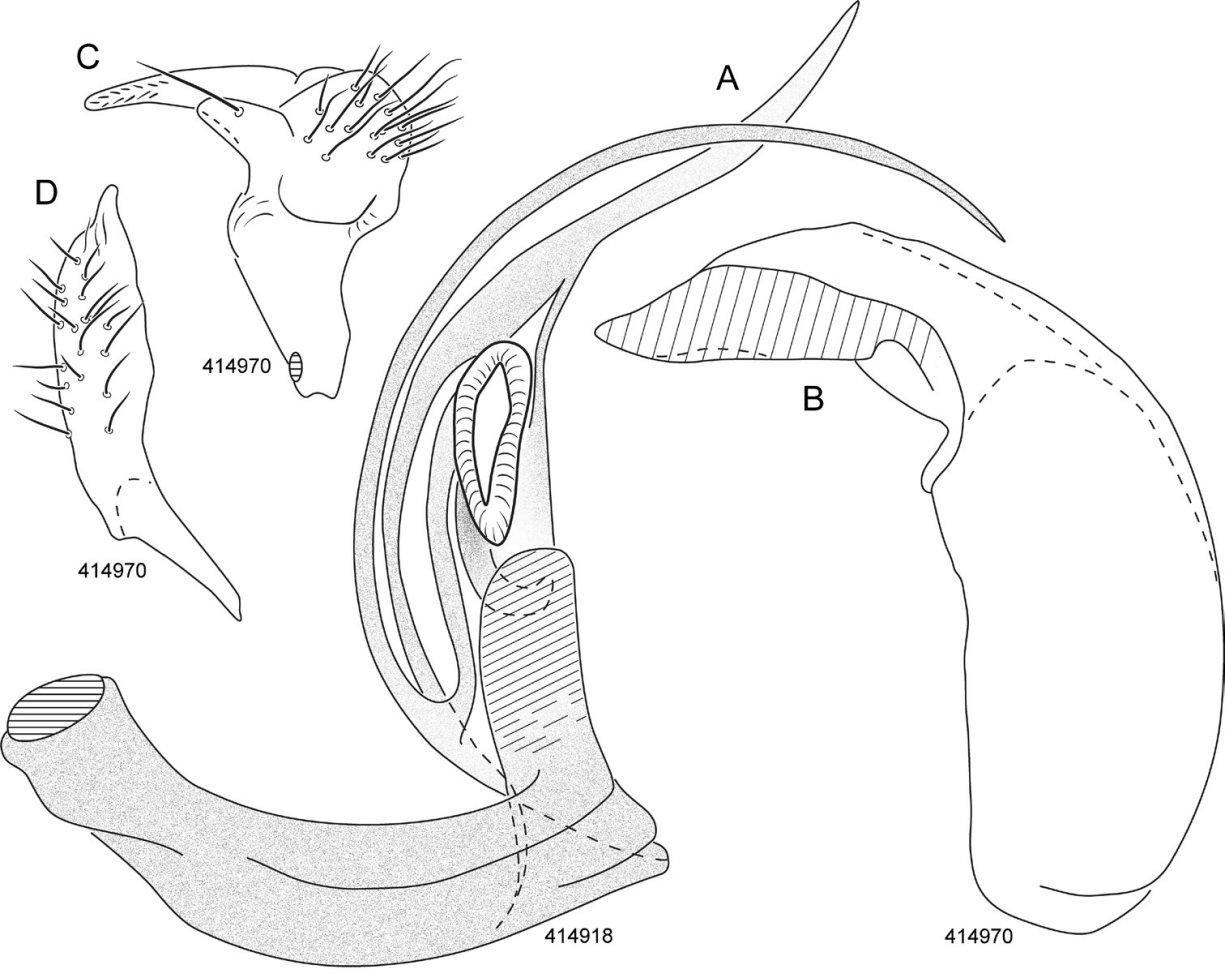
Male genitalia, *Henryognathusthomasi*. **A** Endosoma **B** Phallotheca **C** Left paramere **D** Right paramere.

Recognized by the elongate ovoid body, the moderately prognathous head, the pale tibiae with contrasting dark spots at the bases of the dark spines (Fig. 1A, B), the tarsal claws of moderate length, broad at base, bent medially, with a small, flaplike pulvillus just proximad of bend in claw, and the structure of the male genitalia, with elongate, slender, curving apical endosomal spines, the ventral spine conspicuously bifurcating at a point significantly proximal to the secondary gonopore (Fig. 2A). Body form, head shape, and tibial coloration similar to most *Plagiognathus* species, but proximal bifurcation of “ventral” strap of endosoma unlike the condition seen in *Plagiognathus*, as are the longer, more slender, curving apical spines (Schuh 2001: figs 20–33).

#### Description.

Size moderate (Table 1). COLORATION (Fig. 1A, B): Pale in known species.

*Surface and vestiture*: Dorsum weakly granular, smooth, weakly shining. Vestiture of dorsum composed of reclining pale, golden-shining, simple setae.

*Structure*: Hemelytra sloping laterally, corial margins very weakly convex; frons tumid, clypeus visible from above. Tarsal claws similar to those in *Plagiognathus* (Schuh, 2001: figs 37C, 38D), of moderate length, broad at base, bent medially, with a small, flaplike pulvillus just proximad of bend in claw.

*Genitalia* (Fig. 2): Endosoma strongly twisted and bent medially, basal portion of moderate and uniform width; apical spines long, slender; ventral spine conspicuously bifurcating at a point significantly proximal to the secondary gonopore; endosoma with a broad rectangular flange proximal to secondary gonopore; secondary gonopore moderately large and strongly sclerotized, with a projecting sclerite proximally.

*Phallotheca*: L-shaped, with a broad posterolateral opening on distal portion. Left paramere short, anterior process with a strong subapical seta; right paramere elongate, parallel sided over most of length, with a fingerlike apex.

***Female***: Very similar in shape and proportions to male (Fig. 1B). Coloration as in male. Female genitalia: Not examined.

**Table 1.** Measurements of *Henryognathusthomasi*.

**Species**		**Length**						**Width**				
		**Body**	**CunClyp**	**Head**	**Pron**	**Scut**	**Cun**	**Head**	**Pron**	**Scut**	**InterOc**	**AntSeg2**
♂ **(N = 12)**	**Mean**	**3.73**	**2.56**	**0.30**	**0.45**	**0.45**	**0.55**	**0.73**	**1.04**	**0.53**	**0.34**	**1.27**
	SD	0.23	0.16	0.04	0.03	0.03	0.03	0.02	0.05	0.04	0.01	0.09
	Range	0.83	0.56	0.13	0.11	0.11	0.11	0.09	0.18	0.12	0.03	0.27
	Min	3.41	2.34	0.23	0.41	0.41	0.51	0.68	0.95	0.48	0.32	1.15
	Max	4.24	2.90	0.36	0.52	0.52	0.62	0.76	1.13	0.60	0.36	1.41
♀ **(N = 6)**	**Mean**	**3.93**	**2.77**	**0.39**	**0.51**	**0.48**	**0.51**	**0.75**	**1.11**	**0.57**	**0.38**	**1.28**
	SD	0.09	0.07	0.03	0.03	0.02	0.02	0.02	0.05	0.03	0.02	0.09
	Range	0.21	0.17	0.08	0.08	0.06	0.05	0.04	0.14	0.06	0.04	0.25
	Min	3.83	2.66	0.35	0.47	0.45	0.49	0.73	1.05	0.54	0.36	1.16
	Max	4.04	2.84	0.43	0.55	0.51	0.54	0.77	1.19	0.60	0.41	1.41

#### Etymology.

A combination of Henry (Thomas J. Henry) and -gnathus, from the Greek gnathos, jaw, in reference to the similarity with species of *Plagiognathus*.

#### Discussion.

*Henryognathus* falls within the diagnosis of *Plagiognathus* Fieber, as rendered by Schuh (2001), with the exception of the structure of the male genitalia.

### Henryognathus thomasi sp. n.

Taxon classificationAnimaliaHemipteraMiridae

http://zoobank.org/1B8709C8-6708-4951-B2F2-E621FD40789F

Figures 1 , 2 , Table 1

#### Diagnosis.

Recognized by the moderate size, pale, yellow to yellow-orange coloration in preserved specimens (Fig. 1A, B.), the antennae black in male except segment 1 pale on basal half and with pale apical annulus (Fig. 1A, B), sometimes with central 2/3 of segment 2 pale; and by the structure of the male genitalia (Fig. 2). Among North American taxa most easily confused with species of *Americodema* and *Occidentodema* based on pale coloration and head shape, but lacking the black stripe on the dorsal surface of metafemur as found in those taxa, and pale *Plagiognathus* species (e.g., *P.luteus* Knight), but easily distinguished by its longer, slender, curving apical spines on endosoma, as opposed to the bladelike apical spines in *Plagiognathus* (Schuh 2001: figs 20–33).

#### Description.

***Male***: Very elongate ovoid, of moderate size; mean total length 3.73, mean length apex clypeus-cuneal fracture 2.56, mean width across pronotum 1.04 (Table 1).

*Coloration* (Fig. 1A, B): General coloration pale, yellowish or yellow orange, translucent; membrane pale, veins of cells white; antennal segment 1 pale on basal two-thirds, nearly black on distal third with a pale apical annulus with subapical black spine on interior surface, remaining antennal segments entirely black, or segment 2 sometimes pale medially; labium infuscate at apex; legs generally pale, hind femur with weak dark spots distally; tibiae with dark spines on dorsal surface at bases of black spines, hind femur black at femoral articulation.

*Surface and vestiture* (Fig. 1A, B): Dorsum weakly granular, smooth, weakly shining. Vestiture of dorsum with reclining pale, golden-shining, simple setae.

*Structure*: Hemelytra sloping laterally, corial margins very weakly convex; frons tumid, clypeus visible from above; head projecting below eye by diameter of antennal segment 1; labium reaching to at least posterior margin of abdominal sternum 4. Tarsal claws as in generic description.

*Genitalia* (Fig. 2): In addition to attributes in generic description, ventral spine smoothly curving over an arc of nearly 180 degrees, dorsal spine bent but not uniformly curving. Phallotheca L-shaped, with a broad posterolateral opening on distal portion. Left paramere short, anterior process with a strong subapical seta. Right paramere elongate, parallel sided over most of length, with a fingerlike apex.

***Female***: Very similar in shape and proportions to male. Mean total length 3.93, mean length apex clypeus-cuneal fracture 2.77, mean width across pronotum 1.11 (Table 1). Coloration as in male (Fig. 1A, B). Female genitalia: Not examined.

#### Etymology.

Named for Thomas J. Henry.

#### Hosts.

*Arctostaphylospungens* Kunth, *A.viscida* Parry (fig. 1C, D), and *A.* sp. (Ericaceae). We regard the very few specimens labeled as occurring on *Mimosabiuncifera* Benth. (Fabaceae), *Quercusturbinella* Greene (Fagaceae), and *Rhamnuscalifornica* Eschsch. (Rhamnaceae) to represent either sitting records or the result of commingling of specimens in the field. A total of 54 specimens was collected at light or host information was not recorded by the collector.

The habit of feeding on *Arctostaphylos* is shared with other Phylinae such as *Arctostaphylocorisarizonensis* Schuh and Schwartz, *A.manzanitae* (Knight), *Atractotomusschwartzi* Stonedahl, four species of *Ceratopsallus* Schuh (Schuh 2006), the two known species of the orthotyline genus *Melymacra* Schwartz, four species of the mirine genus *Phytocoris* Hahn, and the dicyphine *Tupiocoriskillamae* Schwartz and Scudder. In all of these cases the distribution of the bugs on their hosts can be extremely patchy.

#### Distribution

**Figure 3.**
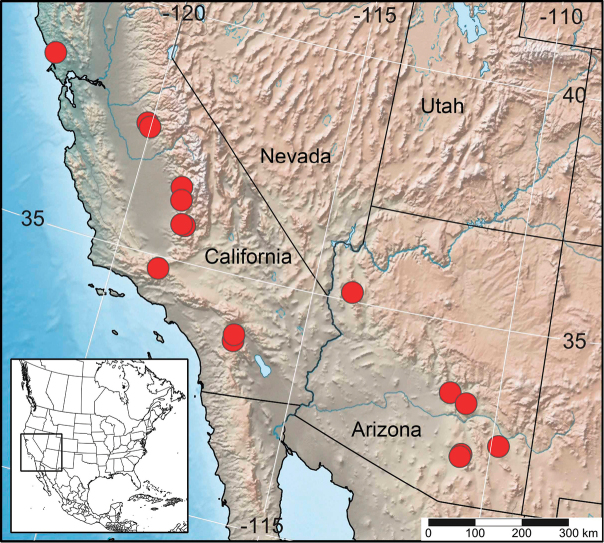
Distribution of *Henryognathusthomasi* in the American southwest.

(Fig. 3): Known from Gila, Graham, Mohave, and Pima counties in Arizona and from Los Angeles, Kern, Mariposa, Riverside, Sonoma, and Tulare counties in California.

**HOLOTYPE**: **USA: California: Tulare Co**.: NE of Springville on Bear Creek Rd near Scicon, 36.21394°N, 118.7716°W, 700 m, 23 May 2004, R.T. Schuh, Cassis, Schwartz, Weirauch, Wyniger, Forero, *Arctostaphylosviscida* Parry (Ericaceae), det. A. Sanders UCR140624, 1♂ (AMNH_PBI 00321005) (AMNH).

**PARATYPES**: **USA: Arizona: Gila Co.**: 8 mi SW jct Rts 87 and 188 (off Rt 87), Tonto National Forest, 33.55989°N, 111.21341°W, 1219 m, 27 May 1983 - 28 May 1983, R. T. Schuh and G. M. Stonedahl, 1♂ (AMNH_PBI 00414940) *Arctostaphylospungens* Kunth (Ericaceae), 3♂ (00414935-00414937), 2♀ (00414938, 00414939) (AMNH). Old CCC Campground S of Globe on Pioneer Pass Rd, 33.39417°N, 110.78583°W, 1433 m, 30 May 1983 - 31 May 1983, R.T. Schuh, G.M. Stonedahl and B.M. Massie, 21♂ (00414898-00414918), 10♀ (00414925-00414934) (AMNH), 1♂ (00414892), 1♀ (00414919) (CAS), 1♂ (00414893), 1♀ (00414920) (CNC), 1♂ (00414894), 1♀ (00414921) (UCB), 1♂ (00414895), 1♀ (00414922) (UCR), 1♂ (00414896), 1♀ (00414923) (USNM), 1♂ (00414897), 1♀ (00414924) (ZISP). **Graham Co.**: Pinaleno Mountains, Stockton Pass, 32.64083°N, 109.84306°W, 1631 m, 01 Jun 1983 - 02 Jun 1983, R. T. Schuh and G. M. Stonedahl, 1♀ (00414944) (AMNH). **Mohave Co.**: Hualapai Mountains, SE of Kingman, T20N R15W, 35.18944°N, 114.05222°W, 1585 m, 09 Jun 1983 - 10 Jun 1983, R. T. Schuh, M. D. Schwartz, G. M. Stonedahl, *Quercusturbinella* Greene (Fagaceae), 1♀ (00414943) (AMNH). **Pima Co.**: 4 mi N of Coronado Natl. Forest boundary on Mount Lemmon Rd, 32.36°N, 110.7°W, 1219 m, 11 Jun 1983, R.T. Schuh, Schwartz, and Stonedahl, *Mimosabiuncifera* Benth. (Fabaceae), 1♂ (00414942) (AMNH). 7.5 mi S of Coronado Natl. Forest boundary on Mount Lemmon Rd, 32.31°N, 110.72°W, 1433 m, 11 Jun 1983, R.T. Schuh, Schwartz, and Stonedahl, 1♂ (00414941) (AMNH). **California: Kern Co.**: 7 km W of Wofford Heights on Rt 155, 35.725°N, 118.52555°W, 1520 m, 26 Jul 1999, M.D. Schwartz, *Arctostaphylos* sp. (Ericaceae), 5♂ (00414945-00414949), 10♀ (00414956-00414965) (AMNH), 1♂ (00414950), 1♀ (00414953) (CNC), 1♂ (00414951), 1♀ (00414954) (UCR), 1♂ (00414952), 1♀ (00414955) (USNM). Cedar Creek Campground on Rt 115, 35.73726°N, 118.61183°W, 1500 m, 26 Jul 1999, M.D. Schwartz, *Arctostaphylos* sp. (Ericaceae), 5♂ (00414966-00414970), 4♀ (00414971-00414974) (AMNH). **Los Angeles Co.**: Tanbark Flats, 33.69111°N, 116.67056°W, 25 Jun 1952, A.A. Grigarick, 1♂ (00125674) (UCD). **Mariposa Co.**: NW of Mariposa off Rt 140 on Bear Valley Rd, 37.57111°N, 120.13243°W, 663 m, 25 May 2004, R.T. Schuh, Cassis, Schwartz, Weirauch, Wyniger, Forero, *Arctostaphylosviscida* Parry (Ericaceae), det. A. Sanders UCR140623, 3♂ (00321062-00321064), 1♀ (00321065), 2♂ (00321066, 00321067), 2♀ (00321068, 00321069) (AMNH). W of Mariposa near Mt. Bullion, 37.49936°N, 120.0435°W, 675 m, 25 May 2004, R.T. Schuh, Cassis, Schwartz, Weirauch, Wyniger, Forero, *Arctostaphylosviscida* Parry (Ericaceae), det. Field ID, 88♂ (00320031- 00320093, 00414993-00415017), 41♀ (00320094-00320103, 00415036-00415065, 00415067) (AMNH), 3♂ (00414975-00414977), 3♀ (00415018-00415020) (CAS), 3♂ (00414978-00414980), 3♀ (00415021-00415023) (CNC), 3♂ (00414981-00414983), 3♀ (00415024-00415026) (UCB), 3♂ (00414984-00414986), 3♀ (00415027-00415029) (UCR), 3♂ (00414987-00414989), 3♀ (00415030-00415032) (USNM), 3♂ (00414990- 00414992), 3♀ (00415033-00415035) (ZISP). **Riverside Co.**: San Jacinto Mountains, 33.81446°N, 116.67918°W, 21 Jul 1929, R. H. Beamer, 2♂ (00292384, 00292385) (KU). San Jacinto Mountains, Pinon Flat, 34.74417°N, 118.89722°W, 28 May 1940, C. D. Michener, *Arctostaphylos* sp. (Ericaceae), 2♂ (00081128, 00081132) (AMNH), 3♂ (00081129-00081131) (UCB). **Sonoma Co.**: Occidental, 38.4075°N, 122.94722°W, 16 Aug 1938, R. I. Sailer, 1♂ (00292386) (KU). **Tulare Co.**: Mineral King Rd E of Three Rivers, 36.47356°N, 118.8465°W, 492 m, 24 May 2004, R.T. Schuh, Cassis, Schwartz, Weirauch, Wyniger, Forero, *Arctostaphylosviscida* Parry (Ericaceae), det. Field ID, 1♂ (00321049), 12♀ (00321050-00321061) (AMNH). NE of Springville on Bear Creek Rd near Scicon, 36.21394°N, 118.7716°W, 700 m, 23 May 2004, R.T. Schuh, Cassis, Schwartz, Weirauch, Wyniger, Forero, *Arctostaphylosviscida* Parry (Ericaceae), det. A. Sanders UCR140624, 11♂ (00320996-00321004, 00321006, 00321007), 29♀ (00321014-00321042) *Rhamnuscalifornica* Eschsch. (Rhamnaceae), det. A. Sanders UCR140642, 2♂ (00321043, 00321044), 4♀ (00321045-00321048) (AMNH), *Arctostaphylosviscida* Parry (Ericaceae), det. A. Sanders UCR140624, 1♂ (00320990), 1♀ (00321008) (CAS), 1♂ (00320991), 1♀ (00321009) (CNC), 1♂ (00320992), 1♀ (00321010) (UCB), 1♂ (00320993), 1♀ (00321011) (UCR), 1♂ (00320994), 1♀ (00321012) (USNM), 1♂ (00320995), 1♀ (00321013) (ZISP).

#### Other specimens examined.

**USA: California: Mariposa Co.**: W of Mariposa near Mt. Bullion, 37.49936°N, 120.0435°W, 675 m, 25 May 2004, R.T. Schuh, Cassis, Schwartz, Weirauch, Wyniger, Forero, *Arctostaphylosviscida* Parry (Ericaceae), det. Field ID, 1 nymph (00415068) (AMNH).

## Discussion

See diagnosis.

## Supplementary Material

XML Treatment for Henryognathus

XML Treatment for Henryognathus thomasi
